# ICU Delirium Is Associated with Cardiovascular Burden and Higher Mortality in Patients with Severe COVID-19 Pneumonia

**DOI:** 10.3390/jcm12155049

**Published:** 2023-07-31

**Authors:** Mateusz Gutowski, Jakub Klimkiewicz, Andrzej Michałowski, Michal Ordak, Marcin Możański, Arkadiusz Lubas

**Affiliations:** 1 Department of Anesthesiology and Intensive Care, Military Institute of Medicine—National Research Institute, 04-141 Warsaw, Poland; jklimkiewicz@wim.mil.pl (J.K.); amichalowski@wim.mil.pl (A.M.); mmozanski@wim.mil.pl (M.M.); 2 Department of Pharmacotherapy and Pharmaceutical Care, Faculty of Pharmacy, Medical University of Warsaw, 02-097 Warsaw, Poland; michal.ordak@wum.edu.pl; 3 Department of Internal Diseases Nephrology and Dialysis, Military Institute of Medicine—National Research Institute, 04-141 Warsaw, Poland; alubas@wim.mil.pl

**Keywords:** delirium, COVID-19, ICU

## Abstract

Background: COVID-19 can lead to functional disorders and complications, e.g., pulmonary, thromboembolic, and neurological. The neuro-invasive potential of SARS-CoV-2 may result in acute brain malfunction, which manifests as delirium as a symptom. Delirium is a risk factor for death among patients hospitalized due to critical illness. Taking the above into consideration, the authors investigated risk factors for delirium in COVID-19 patients and its influence on outcomes. Methods: A total of 335 patients hospitalized due to severe forms of COVID-19 were enrolled in the study. Data were collected from medical charts. Results: Delirium occurred among 21.5% of patients. In the delirium group, mortality was significantly higher compared to non-delirium patients (59.7% vs. 28.5%; *p* < 0.001). Delirium increased the risk of death, with an OR of 3.71 (95% CI 2.16–6.89; *p* < 0.001). Age, chronic atrial fibrillation, elevated INR, urea, and procalcitonin, as well as decreased phosphates, appeared to be the independent risk factors for delirium occurrence. Conclusions: Delirium occurrence in patients with severe COVID-19 significantly increases the risk of death and is associated with a cardiovascular burden. Hypophosphatemia is a promising reversible factor to reduce mortality in this group of patients. However, larger studies are essential in this area.

## 1. Introduction

Among 72,314 people with COVID-19 in China, 81% of cases are reported to be mild (defined in this study as no pneumonia or mild pneumonia), 14% are severe (defined as dyspnea, respiratory frequency ≥ 30 breaths/min, oxygen saturation ≤ 93%, a ratio of arterial partial pressure of oxygen to fraction of inspired oxygen [PaO_2_/FiO_2_] < 300 mm Hg, and/or lung infiltrates > 50% within 24 to 48 h), and 5% are critical (defined as respiratory failure, septic shock, and/or multiple organ dysfunction syndrome or failure) [1].

Although COVID-19 is primarily a pulmonary disease, emerging data suggest that it also leads to cardiac [2,3], hematologic [4,5], neurologic [6,7,8], renal [9], and other complications. Thromboembolic events also occur in patients with COVID-19, with the highest risk occurring in critically ill patients [10]. 

Delirium is a disturbance of consciousness characterized by acute onset and a fluctuating course of inattention, accompanied by either a change in cognition or a perceptual disturbance. This results in the patient’s decreased ability to receive, process, store, and recall information [11]. Delirium develops over a short period of time, from hours to days. It is usually reversible. Typically, delirium is a consequence of a medical condition, substance intoxication or withdrawal, the use of medication, toxin exposure, or a combination of these factors [12]. Delirium etiologies can be classified into two major categories: (a) direct brain insults, e.g., hypotension, hypoxia, hypercapnia, infarcts, brain hemorrhage, trauma, and drugs, and (b) aberrant stress responses, induced by impaired responses to stressors such as infection, surgical trauma, and anxiety [13]. Hypoxemia and systemic hypoglycemia cause acute brain dysfunction in multiple regions. This may lead to impairments in attention and cognition, which meet the criteria for delirium [12]. Localized energy deprivation—for example, by thrombosis or hemorrhage in brain regions critical to attentional processes, such as the caudate nucleus or frontal cholinergic pathways—may also cause delirium [14,15]. The use of sedating medications in critically ill patients, especially sedative hypnotics and anticholinergic agents, is associated with the development of delirium [16]. Another potential factor contributing to the occurrence of ICU delirium during the SARS-CoV-2 outbreak was social isolation, created by “social distancing” strategies and quarantines, which may have proven especially difficult in older adults, with limited support from caregivers [17]. Septic shock is another typical clinical situation where delirium commonly appears. It was shown that this state is followed by severe white matter injury, possibly because of decreased cerebral perfusion or increased blood–brain barrier (BBB) permeability [17]. Acute mental status changes are also linked to primary CNS diseases such as meningitis or encephalitis. There are significant direct brain injury contributors [18]. These and other types of direct brain injury include loss of energy, metabolic disruption, or direct injury to the brain parenchyma, which have subsequent consequences for neurotransmission [19]. Thus, there is no single mechanism triggering delirium, although certain mechanisms may occur more commonly because of increased vulnerability. Behavior alterations occurring during immune stimulation appear to be coordinated by the CNS synthesis of pro-inflammatory mediators. Several routes exist by which a systemic inflammatory signal can be transduced into the brain without compromising the BBB. It is well established that pathogens or pathogen-induced circulating inflammatory mediators can (a) interact directly with neurons in the circumventricular organs, which lack a patent blood–brain barrier; (b) activate the endothelial cells of the brain vasculature to secrete soluble prostaglandins into the brain parenchyma; or (c) activate afferents of the vagus nerve and thus stimulate brain centers by a neural route. It has been shown that the degree of brain vascular endothelial and perivascular cell activation in human post-mortem brains is correlated with the degree of systemic inflammation [18]. In addition, the blood–brain barrier exhibits structural and functional changes with aging [19], diabetes [20], and in Alzheimer’s disease and vascular dementia [21], and this may inappropriately increase the strength of inflammatory signaling.

There are not many pharmaceutical alternatives that have proven effective in managing delirium after its onset. The use of antipsychotics for the prevention or treatment of delirium is not currently supported by evidence [22]. The use of dexmedetomidine for the prevention of delirium is controversial. According to a Cochrane study, dexmedetomidine may reduce delirium’s duration, the time of mechanical ventilation, and the ICU stay. No evidence of a difference in coma-free or delirium-free days, long-term cognitive impairment, or mortality was found in the above meta-analysis [23]. Drugs such as statins, ketamine, melatonin, and ramelton [24] appear to be promising; however, to date, no clear benefits for patients have been shown and more research is needed [25]. The current management of ICU delirium is based on early detection and prevention. Prevention techniques include risk factor control, e.g., early removal of catheters and promoting a comfortable sleeping environment.

The ABCDEF bundle comprises the following: Assess, prevent, and manage pain; Both spontaneous awakening trials and spontaneous breathing trials; Choice of analgesia and sedation (such as avoidance of benzodiazepine-based sedation); Delirium—assess, prevent, and manage; Early mobility and exercise; Family engagement and empowerment. It represents the best management strategy for critically ill patients with delirium [25]. The use of the ABCDEF bundle, which can help to reduce delirium’s frequency in the ICU via a synergistic approach to the patient, may be beneficial. The current management strategy emphasizes early detection and prevention [26,27]. 

This study aimed to investigate risk factors for delirium among COVID-19 patients and its impact on mortality. Our primary goals were to investigate whether delirium was a significant problem in our patient population and whether it affected in-hospital mortality. Secondary outcomes were to identify risk factors in the form of comorbidities and laboratory test results.

## 2. Materials and Methods

A retrospective, cohort study was conducted in the COVID-19 temporary hospital of the Military Medical Institute. The COVID-19 hospital was developed to separate COVID-19 patients. It comprised 48 high-dependency beds (HDU) and 12 intensive care beds (ICU). All patients admitted to the COVID-19 hospital had severe manifestations of the disease, with profound hypoxemia, needing at least a simple oxygen mask for treatment. All COVID-19 patients hospitalized in the COVID-19 hospital between 1 March 2021 and 24 June 2021 were enrolled in the study.

Patients admitted to COVID Hospital received treatment according to polish guidelines. Guidelines were updated many times during the timeframe of study to followed national trends, especially when new drugs proved efficacy in COVID-19. According to the guidelines all patients with pneumonia and need for oxygen supplementation received 6 mg dexamethasone, and low molecule weight heparin. If patient specific criteria of time frame antiviral drug were prescribed if no contraindicated. Patients with progression of ARDS, with severe impairment of lung function received 3 days course of methylprednisolone and tocilizumab. Also intravenous fluids and antipyretics were administrated.

If Acute Respiratory Distress Syndrome (ARDS) resulted with critical Ventilation/Blood Flow mismatch (V/Q mismatch) and mechanical ventilation was initiated we used propofol and fentanyl titrated to achieve level of deep sedation Richmond Agitation-Sedation Scale (RASS) -4. When prone position was used we administered neuromuscular blocking agent. As severe shortage of relaxant was observed patients received infusion of rocuronium, atracurium or cisatracurium. The only criterion used was availability. Such sedation protocol was used as long as possible. Usually after 3 to 5 days due to hypertriglyceridemia we switch from propofol to midazolam with ketamine. When weaning from ventilator was initiated propofol, midazolam, fentanyl, ketamine was tapered down and dexmedetomidine infusion was initiated. Dexmedetomidine was given until extubation, then the dose was reduced 50% every day until infusion was stopped.

Data on medical history were collected from the anamnesis—information on the previous course of the disease, chronic diseases, state of consciousness, respiratory, circulatory and urinary system efficiency. Delirium was diagnosed on the basis of medical observations in the form of acute onset of disturbance of consciousness, fluctuating course of inattention, accompanied by either a change in cognition or a perceptual disturbance.

Basing on electronic medication sheet, we investigated, whether before the onset of delirium patients were prescribed medications with centrally mediated effect: opioids (morphine, fentanyl), non-opioid analgesics (paracetamol, metamizole, dex/ketoprofen), benzodiazepines (midazolam, diazepam). Hydroxyzine was administered to patients in the evening to fight the insomnia. Dexmedetomidine was used among ICU patients, after weaning from deep sedation. This mean that dexmedetomidine usually was administered before the onset of delirium. Quetiapine and haloperidol were administered therapeutically for delirium.

### Statistical Analysis

The medical histories of the enrolled individuals were retrospectively analyzed. Laboratory tests were evaluated on the first day of delirium or at the mean time of delirium occurrence (i.e., 6th day).

Data were presented as means with standard deviations and medians with extremes. Nominal variables were shown as numbers with percentage occurrence. The Kolomorgov–Smirnov test was used to determine the normality of the distribution. Differences between continuous variables with a normal distribution were checked with the *t*-test; otherwise, the Mann–Whitney test was used. Differences between nominal variables were checked with the Chi^2^ test or the Fisher exact test if the group quantity was below 10. Univariable logistic regression and multivariable backward regression analyses were performed to estimate factors with a substantial influence on the observed phenomenon. All analyses were conducted on the raw data, in contrast to multivariable logistic regression, requiring the compensation of data loss with means.

In all tests, a double-sided *p* < 0.05 was considered significant. Statistical analysis was performed using the IBM SPSS Statistics v. 25 (IBM Corp., Armonk, NY, USA) and Tibco Statistica v. 13.3 (TIBCO Software Inc., Greenwood Village, CO, USA) software packages.

## 3. Results

We analyzed 335 patients (age 65.9 ± 15.2 years; 141 of female and 194 of male gender). Demographic information and medical history are provided in Table 1.

Among 335 investigated patients, 72 (21.5%) developed delirium. The onset time was day 5.7 ± 7.2 (median 2.0; min. 1.0; max. 30.0) of hospitalization. The mortality rate was significantly higher in the delirium group, compared to individuals without delirium (59.7% vs. 28.5%; *p* < 0.001). In the logistic regression analysis, delirium increased substantially the risk of death, with an OR of 3.71 (95% CI 2.16–6.89; *p* < 0.001). Patients with delirium were older (76.1 ± 13.7 vs. 63.1 ± 14.4 years; *p* < 0.001), tended to be female (58.3% vs. 37.6%; *p* = 0.002), and more frequently were hospitalized in the HDU (88.9% vs. 76.0%; *p* = 0.022) than in the ICU. Differences in demographic data and medical history among patients with and without delirium during the hospital stay are presented in Table 2.

Comorbidities significantly differed between groups. Discrepancies between the delirium and non-delirium groups were found in the frequency of coronary artery disease (25.0% vs. 13.7%; *p* = 0.021), myocardial infarction (15.3% vs. 4.9%; *p* = 0.003), and atrial fibrillation (30.6% vs. 11.0%; *p* < 0.001). Substantial differences between the groups were observed in the laboratory results. Individuals from the delirium group had higher mean ALT (81.9 ± 246.8 vs. 63.8 ± 52.1; *p* = 0.02), CRP (11.2 ± 8.1 vs. 7.6 ± 10.8; *p* < 0.001), INR (1.7 ± 1.8 vs. 1.2 ± 0.2; *p* < 0.001), PCT (6.20 ± 16.85 vs. 1.86 ± 5.14; *p* = 0.048), creatinine (1.6 ± 1.7 vs. 1.5 ± 2.0; *p* = 0.026), and urea (89.9 ± 67.5 vs. 67.6 ± 50.0; *p* = 0.001) levels. However, the mean phosphate concentration (3.6 ± 1.0 vs. 4.9 ± 2.1; *p* = 0.016) and PLT count (250.6 ± 125.2 vs. 284.9 ± 112.5; *p* = 0.04) were lower in the delirium group compared to the non-delirium group.

A comparison of the comorbidities and laboratory data in both groups is presented in Table 3.

In the univariable logistic regression analysis, comorbidities as well as laboratory results from the estimated day of delirium onset were significantly correlated with delirium occurrence (Table 4).

Then, we performed multivariable backward logistic regression, including data significantly associated with delirium in univariable analyses. The analysis showed that age, chronic atrial fibrillation, high INR, urea, and procalcitonin with low phosphates were independently related to delirium occurrence (Table 5 and Table 6).

Finally, we analyzed the administration of medication with CNS activity. Results are shown in Table 7.

The results showed no correlation between analgesic treatment (paracetamol, metamizole, ketoprofen, tramadol) and the occurrence of delirium. In the delirium group, the more frequent administration of morphine (36.11% vs. 7.22%; *p* < 0.001), quetiapine (31.94% vs. 8.37%; *p* < 0.001), and haloperidol (27.78% vs. 5.32%; *p* < 0.001) was observed. In contrast, the prescription of fentanyl was found to be less frequent at 8.45% vs. 19.39%, *p* = 0.03.

## 4. Discussion

Delirium is a significant independent predictor of death, a prolonged length of stay, hospital readmission, and long-term cognitive impairment in hospitalized patients [28,29,30]. This is also true for the ICU setting. Delirium observed during an ICU stay has been independently linked with greater 6-month mortality, longer hospital stays, and longer post-ICU stay [30]. Delirium has serious adverse effects on mortality, functional outcomes, lengths of stay and institutionalization [25]. In elderly individuals, in addition to in-hospital mortality, it also affects increased mortality and morbidity after discharge from the hospital [31].

Acute brain dysfunction, symptomatically presenting as delirium (also called encephalopathy), may be a feature of the neuro-invasive potential of SARS-CoV-2. The neurotropism of *Coronaviridae* has been demonstrated during the SARS and MERS epidemics [32,33,34]. Given the fact that SARS-CoV and SARS-CoV-2 are similar in terms of pathogenicity, it is quite likely that SARS-CoV-2 has a similar ability to cause delirium [35]. Most *Coronaviridae* share a common viral structure, infection potential, and neurotropism [36,37]. In one series of 183 children hospitalized with acute encephalitis, 12% were associated with *Coronaviridae* infection [38]. There are several mechanisms of coronavirus-related brain damage. One of them relates to the dysfunction of the renin–angiotensin system in the brain. ACE is the major component of the cerebral renin–angiotensin system and is localized in the endothelia of the cerebral vasculature [39]. Circulating renin–angiotensin components do not affect the brain due to the tight blood–brain barrier. However, the general inflammatory response to virus infection impairs BBB integrity, leading to the massive infiltration of renin–angiotensin components into the brain [40]. The uncontrolled infiltration of the brain by renin–angiotensin components induces neuroinflammatory cascades, resulting in extensive neurodegeneration followed by cognitive dysfunction [41]. The SARS-CoVs can enter the brain via the BBB angiotensin-converting enzyme receptors and induce neurodegeneration, astrogliosis, and neuroinflammation. It is noteworthy that SARS-CoV particles have been found in the brain [32,33]. The inflammatory response of the CNS to viral infection seems to be another important reason for poor neurological outcomes and the occurrence of delirium. A few hours after infection, neutrophils and monocytes infiltrate the CNS, and neutrophils seem to be crucial in the disruption of BBB permeability [42,43,44]. A postmortem study documented the massive infiltration of the brain by immune cells, which was associated with neuronal edema and scattered red degeneration [33]. Notably, activated macrophages and microglia have been found in areas of demyelination and play a critical role in myelin destruction [44]. A large amount of damaged myelin following neuroinflammation is potentially immunogenic and activates macrophages again, which initiates a vicious cycle sustaining further inflammation.

The neuroinvasive character of COVID-19 may manifest by neurological symptoms. Possible neurological manifestations of COVID-19 include: toxic/metabolic encephalopathy, stroke, seizures, and hypoxic/ischemic brain injury [45]. There is also a growing body of evidence reporting of long COVID presentation [46].

Studying delirium among COVID-19 patients needs paying attention on sepsis-associated encephalopathy (SAE). SAE shares common paths with mechanisms of SARS-CoV-2 mediated CNS injury: an intense inflammatory process, microcirculation disorders, blood-brain barrier compromise, microscopic brain injury.

SAE is commonly seen among critically ill patients. It is characterized by diffuse cerebral dysfunction that follows sepsis in the absence of a direct CNS infetion, anatomical abnormality, or another type of encephalopathy (such as hepatic or renal encephalopathy). Patients with SAE have signs of severe systemic infection.

SAE presents as a spectrum of altered brain function from delirium to coma. As mortality increases with severity of SAE, the early identification and management of patients with SAE is critical to reduce morbidity and mortality. Delirium is often the first manifestation of sepsis.

It is important to identify delirium occurrence among ICU patients as an initial feature of SAE, and aggressive investigation and treatment of the infection and associated systemic effects, such as hypotension, is the most effective strategy to reduce morbidity and mortality associated with the disease [47,48,49].

In our study, we examined 335 patients with severe COVID-19, of whom 72 developed delirium. Among patients with delirium, mortality was 59.7%, compared to 28.5% in those without delirium (*p* < 0.001).

Our data are consistent with the results from other studies. Rebora et al. concluded that delirium was prevalent and associated with in-hospital mortality among older patients hospitalized with SARS-CoV-2 infection [50]. Kotfis et al. reported that the odds of mortality in patients with COVID-19 presenting with delirium during a hospital stay was over seventeen times higher compared to patients without delirium. In critical illness, such as severe SARS-CoV-2 infection, delirium is an early and often the only sign of the deterioration of homeostasis and should be monitored and prevented to avoid increased mortality [51]. The higher prevalence of delirium among HDU patients, compared to ICU patients, may be explained by the fact that delirium can be diagnosed among conscious patients. In the ICU, delirium is diagnosed only among individuals with reduced sedatives. Many ICU patients die while deeply sedated and paralyzed.

Our results showed that markers of impaired kidney function (urea, creatinine), liver injury (ALT, INR), and inflammation (CRP, PCT) significantly differed between the delirium and non-delirium groups. Interestingly, the delirium group had a lower concentration of serum phosphates than the group without delirium.

The presented results confirm the effect of generalized inflammation caused by COVID-19 on the functioning of numerous organs, including the central nervous system, which gives clinical symptoms in the form of delirium [52].

We found that comorbidities such as CAD, MI, AF, and pre-existing dementia were more frequent in the delirium group. Mendes et al. reported that the main risk factor for delirium occurrence was pre-existing dementia [53]. Wilke et al. pointed out a neurodegenerative disease history [54].

In the presented study, we found that lower concentration of phosphates is an independent risk factor for delirium. Hypophosphatemia in sepsis may result from: poor nutrition, steroid therapy, vitamin D deficiency, inappropriate fluid therapy, refeeding syndrome, diuretics, insulin, catecholamines [55]. An interesting observation is that 80% of septic patients have hypophosphatemia associated with elevated concentrations of inflammatory cytokines such as tumor necrosis factor (TNF) alpha and interleukin (IL)-6 and other inflammatory cytokines included in cytokine storm in COVID-19 [56,57]. Studies concerning impact of phosphate concentrations on mortality are inconclusive [58,59]. Our results suggest a link between hypophosphatemia and delirium and indirectly with mortality, as shown by studies in 6190 patients where hypophosphatemia is an independent risk factor for sepsis mortality [58]. However, there are studies suggesting that hyperphosphatemia correlates with a poorer prognosis [59]. Hypophosphatemia observed among patients with severe COVID-19 may be secondary to malnutrition. Also risk factors for hypophosphatemia as steroid therapy [60], vitamin D [61] deficiency are common in COVID-19 patients. Further research might be beneficial to better understand these relationships.

Pain treatment in our patients was carried out according to good clinical practice based on the WHO analgesic ladder [62,63]. A priori, the COVID-19 patients did not receive pain treatment beforehand. Analgesic treatment was initiated when patients reported symptoms to medical staff. The pain was treated in proportion to the symptoms. In mild pain, non-opioid analgesics (i.e., paracetamol, metamizole, NSAIDs (ketoprofen)) was used. According to protocol, paracetamol 4g/day was used for pain and fever control; if there was no improvement, metamizol was added. If this was not sufficient, tramadol 200 mg/day was added [64]. None of the patients were prescribed morphine due to pain.

Patients who had persistent dyspnea symptoms that persisted with adequate oxygen therapy received morphine infusion. In the ICU, intubated patients received deep sedation based on propofol and fentanyl. If the desired sedation level was not achieved, midazolam was added. In the case of severe hypertriglyceridemia, propofol was switched to midazolam with ketamine. Deep sedation was used to facilitate an advanced mode of ventilation by minimizing ventilation asynchrony [65].

The analysis of medication use in our study showed no correlation between analgesic treatment (paracetamol, metamizole, ketoprofen, tramadol) and the occurrence of delirium. However, patients treated with morphine had a higher incidence of delirium.

Patients who developed delirium were more likely to be treated with morphine. (36.11% vs. 7.22%; *p* < 0.001). This coincides with the view that opioid treatment is an independent factor in the onset of delirium, regardless of pain [66].

In our study, fentanyl was given to patients in the most severe condition—ventilator therapy, CVVHDF, and VV-ECMO. A large proportion of these patients died during intensive treatment, under deep sedation. In the presented analysis, fentanyl use was associated with a lower incidence of delirium (8.45% vs. 19.39%; *p* = 0.030), which may have been a result of the patient selection.

Our patients were given benzodiazepines, namely midazolam and diazepam. Diazepam was used in patients with alcohol withdrawal syndrome. Midazolam has been used in ICU patients to deepen sedation when ventilator ventilation asynchrony is present or there are increased triglycerides in patients sedated with propofol [65]. In high-dependency unit patients, benzodiazepines were avoided [64]. The only exception was terminal sedation, when morphine alone was insufficient to control respiratory distress, fear, and agitation. The supply of benzodiazepine did not significantly affect the occurrence of delirium (18.06% vs. 16.73%; *p* = 0.791). The correlation between the use of quetiapine and haloperidol and the onset of delirium was related to the fact that these drugs were used to treat the delirium that occurred [25,62].

It is also important to explain antimicrobial regimen. No prophylactic antibiotics were used. Empiric antibiotic treatment was prescribed only when clinical findings suggested concomitant bacterial infection. As we found WBC count and CRP not to be useful, we initiated antibiotic treatment basing on PCT level. None of the patients received antibiotic with high potential for neurotoxicity such as cefepime, imipenem, cefoxitin, voriconazole. If empiric treatment with beta lactams was initiated we used ceftriaxone or piperacillin/tazobactam or meropenem. For empiric treatment of suspected gram-positive infection we used vancomycin or linezolid. Noteworthy, some patients need target treatment for NDM infection with colistin and tigecycline for confirmed VRE pneumonia.

Besides promising results, we encountered some limitations in our study. This was a single-center study, and further analyses are needed so that our results can be translated to a wider population. Furthermore, during the pandemic, medical staff were limited. Due to the high volume of patients, we had to suspend some time-consuming measures, such as active screening for delirium, e.g., with CAM-ICU. Delirium is divided into 3 types: hyperactive, hypoactive and mixed [67]. The protocol of our study probably increases the sensitivity of recognizing hyperactive, mixed and behavioral delirium. The main limitation is the retrospective classification of delirium status. Hence, the number of patients with delirium, especially the hypoactive variety, may be underestimated in our study. We analyzed the drugs that patients were taking, but we did not take into account the length of use and dosage of individual drugs, which makes it impossible to precisely analyze the problem of the effect of drugs on delirium, but only signals a possible problem.

## 5. Conclusions

The consequences of SARS-CoV-2 infection go far beyond lung infection. COVID-19 causes generalized inflammation, leading to multiple organ failure. We can indirectly observe this in the form of damage to individual organs, e.g., liver damage as an increase in INR or renal failure due to an increase in urea. Delirium occurrence in patients with severe COVID-19 significantly increases the risk of death and is associated with a cardiovascular burden. Hypophosphatemia is a promising reversible factor to decrease mortality in this group of patients. However, more extensive studies are essential in this area.

## Figures and Tables

**Table 1:** Demographic data and medical history of enrolled patients.

Variable		n	%
Status	Deceased	118	35.2
	Moved to non-COVID-19 ward	73	21.8
	Discharged	114	34,0
Gender	Female	141	42.1
	Male	194	57.9
Ward type	Intensive care unit	71	21.2
	High-dependency unit	264	78.8
Obesity		69	20.6
Malignancy		44	13.1
Hypertension		192	57.3
Chronic kidney disease		43	12.8
Diabetes		85	25.4
Coronary heart disease		54	16.1
Heart failure		37	11.0
History of myocardial Infarction		24	7.2
Chronic atrial fibrillation		51	15.2
Tobacco smoker		27	8.1
Asthma		18	5.4
Chronic obstructive pulmonary disease		17	5.1
Dementia		15	4.5
Delirium onset		72	21.5

**Table 2:** Comparison of demographic data and medical history between patients with and without delirium.

Variable	Delirium		Non-Delirium		Significance *p*
	(n = 72)		(n = 263)		
	N	%	n	%	
Deceased	43	59.7%	75	28.5%	<0.001

Gender (female)	42	58.3%	99	37.6%	0.002

Ward (HDU)	64	88.9%	200	76.0%	0.022
Obesity	11	15.3%	58	22.1%	0.208
Malignancy	7	9.7%	37	14.1%	0.432
Hypertension	44	61.1%	148	56.3%	0.462
Chronic kidney disease	13	18.1%	30	11.4%	0.135
Diabetes	22	30.6%	63	24.0%	0.254
Coronary artery disease	18	25.0%	36	13.7%	0.021
Heart failure	9	12.5%	28	10.6%	0.673
History of myocardial infarction	11	15.3%	13	4.9%	0.003
Chronic atrial fibrillation	22	30.6%	29	11.0%	<0.001
Tobacco smoker	5	6.9%	22	8.4%	0.811
Asthma	3	4.2%	15	5.7%	0.773
Chronic obstructive pulmonary disease	3	4.2%	14	5.3%	0.969
Preexisting dementia	6	8.33%	9	3.42%	0.002

**Table 3:** Comparison of comorbidities and laboratory results between groups.

	Delirium (n = 72)		Non-Delirium (n = 263)		Significance *p*
Variable	Mean ± SD	Median (Min, Max)	Mean ± SD	Median (Min, Max)	
Age (years)	76.1 ± 13.7	78 (35, 100)	63.1 ± 14.4	66 (19, 97)	<0.001
HGB [g/dL]	11.9 ± 2.4	11.9 (7.1, 20.1)	12.4 ± 2.2	11.9 (6.4, 16.7)	0.186
Albumin [g/dL]	2.9 ± 0.4	3.0 (2.2, 3.8)	3.3 ± 3.6	2.9 (1.6, 32.7)	0.638
ALT [U/L]	81.9 ± 246.8	39.0 (5.0, 1697.0)	63.8 ± 52.1	50.0 (5.0, 386.0)	0.02
AST [U/L]	99.3 ± 339.4	47.0 (10.0, 2317.0)	45.1 ± 28.2	37.0 (10.0, 163.0)	0.35
CRP [mg/L]	11.2 ± 8.1	10.4 (0.6, 38.9)	7.6 ± 10.8	3.3 (0.1, 58.2)	<0.001
TP [g/dL]	5.9 ± 0.6	6.0 (4.6, 6.8)	5.6 ± 0.7	5.6 (4.8, 7.0)	0.276
Bilirubin [mg/dL]	1.2 ± 3.4	0.5 (0.1, 19.5)	0.5 ± 0.3	0.5 (0.2, 2.2)	0.395
CHOL [mg/dL]	175.8 ± 61.4	168.5 (110.0, 292.0)	170.8 ± 50.5	178.0 (40.0, 302.0)	0.791
LDH [U/L]	674.9 ± 1282.7	387.5 (61.0, 7087.0)	428.6 ± 196.6	386.5 (87.0, 1096.0)	0.708
Ferritin [ug/L]	2064.2 ± 5520.5	825.0 (117.0, 34,243.0)	1928.3 ± 4432.1	1100.0 (45.0, 46,830.0)	0.41
Fibrinogen [mg/dL]	543.6 ± 192.6	538.0 (177.0, 915.0)	470.3 ± 178.9	448.0 (175.0, 993.0)	0.115
Phosphates [mg/dL]	3.6 ± 1.0	3.3 (2.0, 5.6)	4.9 ± 2.1	4.3 (1.7, 12.4)	0.016
INR	1.7 ± 1.8	1.2 (0.8, 11.6)	1.2 ± 0.2	1.14 (0.9; 2,1)	<0.001
Creatinine [mg/dL]	1.6 ± 1.7	1.0 (0.3, 9.8)	1.5 ± 2.0	0.8 (0.3, 13.5)	0.026
Urea [mg/dL]	89.9 ± 67.5	70.0 (25.0, 417.0)	67.6 ± 50.0	47.0 (13.0, 280.0)	0.001
PLT [K/uL]	250.6 ± 125.2	231.5 (46.0, 577.0)	284.9 ± 112.5	278.0 (32.0, 624.0)	0.04
Dimer D [ug/L]	6.3 ± 14.8	1.7(0.3, 87.5)	6.7 ± 15.8	1.6 (0.18, 113.13)	0.717
PCT [ng/mL]	6.20 ± 16.85	0.34 (0.04, 75.42)	1.87 ± 5.14	0.19 (0,03; 31.64)	0.048
WBC [10^9^/L]	11.1 ± 6.5	9.9 (3.41, 46.59)	12.4 ± 7.4	10.7(1.5, 49.0)	0.251

ALT—alanine aminotransferase, AST—asparagine aminotransferase, CRP—C-reactive protein, Chol—total cholesterol, HGB—hemoglobin, INR—international normalized ratio, LDH—lactate dehydrogenase, TP—total protein, PLT—platelets, PCT—procalcitonin, WBC—white blood cells.

**Table 4:** Significant results of univariable regression analysis for delirium occurrence.

Variable	Odds Ratio	95% CI	Significance *p*
Male gender	0.43	0.25–0.73	0.002
Age	1.08	1.05–1.11	<0.001
HDU stay	2.52	1.15–5.54	0.022
Coronary artery disease	2.10	1.11–3.98	0.022
History of myocardial infarction	3.47	1.48–8.12	0.004
Chronic atrial fibrillation	3.55	1.89–6.68	<0.001
C-reactive protein	1.31	1.03–1.06	0.003
Procalcitonin	1.04	1.00–1.09	0.047
Phosphates	0.59	0.37–0.95	0.03
International normalized ratio	16.31	2.62–101.40	0.003
Creatinine	1.04	0.91–1.19	0.588
Urea	1.01	1.00–1.11	0.01
Platelets	0.98	0.99–1.00	0.041

CI—confidence interval.

**Table 5:** Results of the backward stepwise multivariable logistic regression analysis of anamnesis data for delirium occurrence.

Variable	Odds Ratio	95% CI	*p*-Value	AUC	Significance *p* (AUC)
Age	1.07	1.05–1.10	<0.001	0.767	0.033
Chronic atrial fibrillation	2.90	1.06–4.14	0.035		

AUC—area under curve; CI—confidence interval.

**Table 6:** Results of the backward stepwise multivariable logistic regression analysis of laboratory data for delirium occurrence.

Variable	Odds Ratio	95% CI	*p*-Value	AUC	Significance *p* (AUC)
Phosphates	0.62	0.44–0.88	0.008	0.683	0.037
Urea	1.01	1.00–1.01	0.008		
INR	12.60	1.83–86.81	0.010		
Procalcitonin	1.05	1.00–1.10	0.033		

AUC—area under curve; CI—confidence interval.

**Table 7:** Comparison of medication use between patients with and without delirium.

Medication	Delirium		Non-Delirium		Significance *p*
	(n = 72)		(n = 263)		
	n	%	n	%	
Ketoprofen	1	1.39	9	3.42	0.696
Metamizole	4	5.56	15	5.70	1.000
Paracetamol	21	29.17	100	38.02	0.166
Dexmedetomidine	10	13.89	23	8.75	0.194
Tramadol	5	6.94	9	3.24	0.186
Morphine	26	36.11	19	7.22	<0.001
Fentanyl	6	8.45	51	19.39	0.030
BZD	13	18.06	44	16.73	0.791
Hydroxyzine	16	22.22	65	24.71	0.662
Quetiapine	23	31.94	22	8.37	<0.001
Haloperidol	20	27.78	14	5.32	<0.001

BZD—benzodiazepines (midazolam, diazepam).

## Data Availability

The dataset is maintained by the authors and available on request.
